# Recognizing Age-Separated Face Images: Humans and Machines

**DOI:** 10.1371/journal.pone.0112234

**Published:** 2014-12-04

**Authors:** Daksha Yadav, Richa Singh, Mayank Vatsa, Afzel Noore

**Affiliations:** 1 West Virginia University, Morgantown, West Virginia, United States of America; 2 IIIT Delhi, New Delhi, Delhi, India; University of California, San Diego, United States of America

## Abstract

Humans utilize facial appearance, gender, expression, aging pattern, and other ancillary information to recognize individuals. It is interesting to observe how humans perceive facial age. Analyzing these properties can help in understanding the phenomenon of facial aging and incorporating the findings can help in designing effective algorithms. Such a study has two components - facial age estimation and age-separated face recognition. Age estimation involves predicting the age of an individual given his/her facial image. On the other hand, age-separated face recognition consists of recognizing an individual given his/her age-separated images. In this research, we investigate which facial cues are utilized by humans for estimating the age of people belonging to various age groups along with analyzing the effect of one's gender, age, and ethnicity on age estimation skills. We also analyze how various facial regions such as binocular and mouth regions influence age estimation and recognition capabilities. Finally, we propose an age-invariant face recognition algorithm that incorporates the knowledge learned from these observations. Key observations of our research are: (1) the age group of newborns and toddlers is easiest to estimate, (2) gender and ethnicity do not affect the judgment of age group estimation, (3) face as a global feature, is essential to achieve good performance in age-separated face recognition, and (4) the proposed algorithm yields improved recognition performance compared to existing algorithms and also outperforms a commercial system in the *young image as probe* scenario.

## Introduction

Facial images convey a substantial amount of information such as the individual's identity, ethnicity, gender, age, and emotional state [1]. This knowledge plays a significant role during face-to-face communication between humans. Use of facial information during these communications is made possible by the remarkable ability of humans to accurately recognize and interpret faces in real time. Over the past few decades, many automatic face recognition algorithms have been developed. However, it is crucial as well as challenging to develop an algorithm which is robust to variations such as pose, illumination, and expression. Another important challenge of face recognition is matching face images with age variations. Developing age-invariant face recognition algorithms can prove to be beneficial in many applications such as locating missing persons, homeland security, and passport services. In fact, for large-scale applications, adding invariance to aging is a very important requirement.

**Figure 1:**
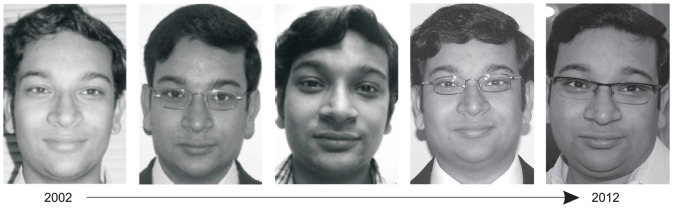
Face images of an individual illustrating variations due to aging across different years.

Aging affects the appearance of a face in diverse ways. It has been observed that every person has a personalized aging pattern depending on numerous factors such as genetics, ethnicity, dietary habits, environmental conditions, and stress level [2], [3]. Further, the process of facial aging is not uniform across time. During formative years of a person, the variations in the shape of a face are more prominent while in the later stages of life, texture variations such as wrinkles and pigmentation are more visible [4], [5]. Fig. 1 shows face images of an individual with age variations.

There are two aspects of building an age-invariant face recognition system: (1) facial age estimation and (2) age-separated face recognition. Accurate age estimation is crucial in a variety of situations such as the need to automatically estimate the age of an individual buying alcohol or cigarettes. In an extensive literature review on age estimation by humans, Rhodes [4] had shown that humans can estimate the age of previously unseen faces quite accurately. However, the proficiency may vary depending on both local and global features. The researchers have examined how adept humans are in estimating the facial age and various aspects that could affect the perceived age. Burt and Perrett [6] evaluated the accuracy of young and old adults in estimating the age of subjects ranging from 20 to 54 years. The study suggests that the predicted age deviated by 2.39 years. Jones and Smith [7] analyzed the influence of local features such as eyes and nose on age estimation. The findings suggest that the eye region is important for age prediction. In an interesting experiment, George and Hole [8] observed that manipulations in the features influence the age estimation precision. The experiments conducted in [9], [10] conclude that even if a region is hidden in the face image, i.e. a source of information is missing, the ability to estimate age is not completely diminished.

The problem of perceived facial age has also been studied by computer vision researchers. Kwon and Lobo [11] are among the first to formulate an age estimation approach based on the facial image. They used anthropometry of the face and facial wrinkle density to classify the input image into three broad categories: infants, young adults, and senior adults. Ramanathan and Chellappa [12] proposed an algorithm to estimate the age gap between a given pair of images. Fu and Huang [13] proposed the use of manifold learning to estimate the age. They applied various manifold learning techniques such as Locality Preserving Projections and Orthogonal Locality Preserving Projections to construct a low-dimensional manifold. Yang and Ai [14] used Local Binary Pattern (LBP) along with AdaBoost.

Some researchers have dedicated their research on finding the effect of group bias on the performance of age estimation. Anastasi and Rhodes [15] observe that age estimation is more precise while predicting the age of images belonging to one's own age group. On the other hand, Burt and Perrett [6] refute the presence of any such own group bias. Anzures et al. [16] analyzed the effect of sociocultural interactions on one's efficiency to estimate the age of the stimuli face. As per their findings, Japanese and Chinese are quicker in their response to estimating the face of East Asian faces than Asian-Canadian participants.

The other aspect of facial aging is face recognition across aging. Lanitis et al. [17], [18] proposed utilizing the training images for finding the relationship between the coded face representation and the facial age of the subject. This relationship is then utilized for estimating the age of a facial image and simulating the facial appearance at any given age. Park et al. [19] developed a 3D facial aging model to address the problem of age-invariant face recognition. Their approach is based on the fact that exact craniofacial aging can be developed only in 3D domain. Li et al. [20] proposed a discriminative model (referred to as DM) for age-invariant recognition. They developed an approach involving the use of scale invariant feature transform (SIFT), multi-scale local binary pattern as local descriptors, and multi-feature discriminant analysis. Guo et al. [21] studied the relationship between face recognition accuracies and age intervals on MORPH-II, a face database. They observe that when the age gap between the gallery and probe images is more than 15 years, the performance decreases much more as compared to within 15 years.

The above mentioned research efforts in computer vision domain suggest that there is a vast scope of improvement in developing automated systems which can mitigate the effect of facial aging. The objective of this research is to study the process of facial aging from the perspective of human cognition and to take cues from it to improve face recognition algorithms. This would enable us to achieve age invariance in facial recognition. Key contributions of this research are listed below.

1. Analyzed human performance for estimating facial age. Specifically, we

- evaluated faces pertaining to different age groups and analyzed facial age estimation results;
- investigated facial cues that are utilized by humans to precisely estimate the age of people belonging to various age groups; and
- analyzed participant's gender, age, and ethnicity bias on the ability to predict age.

2. Studied human performance for recognizing age-separated images. Specifically, we

- determined if humans are able to recognize age-separated images of the same person;
- assessed which local features are employed for the above mentioned face recognition task; and
- evaluated if the cues gathered from the human study can be utilized to develop an age-invariant face recognition algorithm.

3. Developed an algorithm that incorporates the observations obtained by analyzing the results of the first two studies.

- The proposed algorithm first estimates the age group of a probe image. In this research, we have focused on estimating the age group rather than the exact age. This can potentially help in (1) indexing images across ages, and (2) learning important characteristics such as features for every age-group, which can be utilized during automatic face recognition.
- Once the age group is estimated, texture features are extracted for different facial regions. For matching, the weights associated with each facial region captured from human responses are used to combine the information for decision making. The experimental results suggest that incorporating human performance in algorithm enhances the capabilities of automatic face recognition.

To the best of our knowledge, this research is the first to study various aspects of facial aging, analyze human perception of aging facial features, and integrate these findings in an automatic face recognition algorithm.

## Methods

### Ethics Statement

The study was conducted at Amazon's Mechanical Turk (MTurk), which is an online crowd-sourcing platform. At MTurk, only individuals who are above 18 years of age can register and work as participants. We follow the policies of MTurk which clearly transfer the rights of any survey to the requester and the participants are informed of this at the time of their online registration. The participants' consent to fill and submit the survey is taken as their willingness to participate in our study. Further, at the beginning of the study, we also inform the participants that their responses would be used for research and analysis purposes. The images shown in the paper belong to the authors of the paper and they are used for illustrative purposes. The authors in this manuscript have given written consent to publish their images. All the procedures used in the current study are approved by the Indraprastha Institute of Information Technology (IIIT)-Delhi Ethics Board.

### Participants

Amazon's MTurk is a platform that enables researchers to conduct research by offering features such as: a unified participant compensation model, participants having diverse demographics, an efficient procedure of study design, participant enrollment, and data gathering. MTurk allows the researchers or the *requesters* to post tasks such as surveys, studies, and experiments which are, in turn, completed by the participants or *workers*. The participants are paid an amount fixed by the requester upon successful completion of the task. A research conducted by Buhrmester et al. [22] on the effectiveness of MTurk suggests that MTurk not only offers a rich pool of diverse participants but can also be used for economically acquiring large amount of good quality data over a short span of time. In our study, 482 individuals participated, out of which there were,

- 366 Indian adults (Mean Age (M)  = 33.45 years, Standard Deviation in Age (SD)  = 11.67 years, 149 males, 217 females),
- 81 Caucasian adults (M = 35.39 years, SD  = 10.74 years, 43 males, 38 females),
- 29 Asian (non-Indians) adults (M = 28.13 years, SD  = 6.93 years, 6 males, 23 females),
- 3 African adults (M = 30.33 years, SD  = 8.17 years, 2 males, 1 female), and
- 3 participants with undisclosed ethnicity (M = 27.12 years, SD  = 1.7 years, 1 male, 2 females).

The responses from all the participants have been analyzed in the study in order to preserve the diversity in the responses.

### Stimuli

The stimuli faces have been selected from 36 male and 18 female subjects from the FG-Net Facial Aging Database [23] and IIIT-Delhi Facial Aging Database [24], [25]. Out of the total 54 distinct subjects, there are an equal number of Indian and Caucasian subjects. The number of images per subject varies from one to four. The chosen images represent the unconstrained nature of the real world conditions.

For evaluation, 10 sets of assignments are created and one set is randomly assigned to every participant. Each set contains three questions.

**Figure 2:**
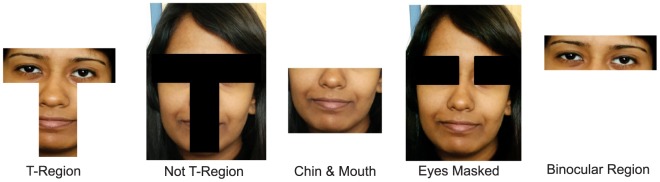
Sample facial regions presented to participants for age group estimation.

**Figure 3:**
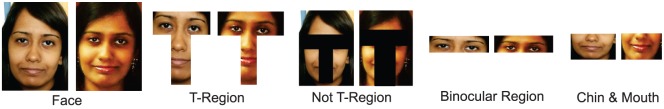
Sample images presented to the participants for recognizing age-separated images of individuals.

1. The first question contains five facial images and the participants are asked to estimate the age group from the given face image. Similar to a previous research [26], the age of face stimuli belongs to one of the following 10 age groups: 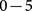, 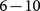, 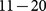, 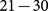, 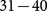, 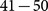, 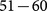, 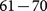, 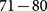, and >80.
2. Five images of various facial regions such as the T-region, binocular region, chin region, eyes portion masked, and T-region masked are shown to the participants. They have to estimate the age group corresponding to every facial part individually. Fig. 2 shows some example images that are presented to the participants belonging to each facial region. These images also belong to one of the 10 earlier mentioned age groups.
3. In the last set of questions, five pairs of age-separated images are shown to the participants and they are asked to determine if the pair of images belongs to the same individual or not. Some sample images are shown in Fig. 3.

### Procedure

Each participant is randomly assigned one of the 10 sets. The participant is supposed to answer the three questions in the Stimuli section. There is no time constraint on the participant to submit the responses. Each participant sees a face image and an identity only once to ensure there is no bias. In all the questions, a mixture of stimuli from different ethnic groups and ages is presented to each participant.

## Results and Discussion

The analysis of responses obtained are classified into four categories and key observations are discussed in this section.

### Age Group Prediction Accuracy

The responses on predicting age group based on the face stimuli presented to participants are summarized in a stimulus-response confusion matrix shown in Table 1. The confusion matrix is used to determine various performance measures of participants to accurately predict the age group category of the face stimulus shown. The humam performance is evaluated in terms of:

**Table 1:** Confusion matrix showing the actual and predicted age groups in the task of age estimation by human participants.

Stimuli Age Group	Predicted Age Group									
	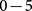	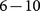	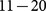	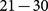	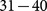	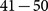	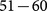	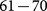	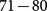	> 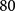
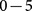	**211**	32	0	1	0	0	0	1	0	0
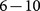	8	**193**	43	2	0	0	0	0	0	0
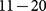	0	1	**121**	104	17	2	1	0	1	0
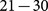	0	2	77	**110**	48	5	0	0	0	0
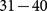	0	1	0	8	**107**	59	12	1	0	0
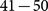	0	0	0	0	17	**63**	51	35	8	0
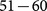	0	0	0	1	6	47	**105**	71	7	0
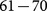	0	0	0	0	0	4	31	**36**	5	0
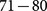	0	0	1	2	3	15	56	80	**52**	15
> 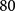	0	0	0	0	0	6	12	12	3	**16**

1. Sensitivity,
2. Specificity,
3. Discriminability index (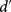), and
4. Information entropy.

*Sensitivity* (or accuracy) represents the true positive performance [27]. However, it alone may not fully represent the performance of participants. We are also interested in the performance of the participants in accurately predicting if a face stimulus does not belong to a particular age group. This information can be obtained from *specificity* [27] which represents the true negative performance. Table 2 summarizes the sensitivity and specificity values for each age group. It shows that the age groups for which the participants were able to best estimate the face stimuli were age groups 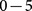 and 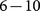 with an accuracy of 86.12% and 78.46% respectively. In contrast, the two lowest age group categories that the participants had difficulty in estimating the face stimulus were age groups 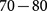 and >80 with accuracies of 23.21% and 32.65% respectively. The specificity for these two age groups is 98.59% and 99.20% respectively indicating that participants are highly confident about a face image not belonging to other age groups. These measures provide valuable insights about age prediction judgments by humans.

**Table 2:** Multiple quantitative measures for analyzing the performance of human participants in estimating age group of visual face stimuli.

	Analysis of Perceptual of Humans in Discrimination of Age Groups by Humans						Automatic Age Group Estimation
Age Group of Face Stimuli	Sensitivity or Accuracy (in %)	Specificity (in %)	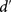	Stimulus Entropy (H(S)) (in bits)	Noise (H(S 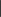 r)) (in bits)	Information Entropy (I(S 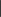 r)) (in bits)	Face++ Accuracy (in %)
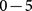	86.12	99.52	2.7500	0.3782	0.0612	0.3171	100
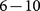	78.46	97.86	2.3971	0.3790	0.1072	0.2718	100
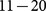	48.99	92.80	1.4453	0.3798	0.1735	0.2063	60
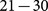	45.46	93.00	1.3422	0.3758	0.1978	0.1780	20
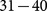	56.92	94.77	1.6776	0.3275	0.1595	0.1680	80
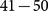	36.21	92.13	1.0658	0.3132	0.2127	0.1005	20
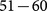	44.30	90.36	1.3085	0.3717	0.2208	0.1509	20
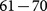	47.37	89.20	1.3981	0.1839	0.1226	0.0613	20
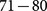	23.21	98.59	0.6292	0.3608	0.2030	0.1578	0
> 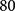	32.65	99.20	0.9543	0.1347	0.0856	0.0491	0

The results show that the accuracy is high when the stimuli faces belong to age groups 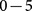 and 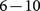.

In response to different visual stimuli, the participants need to make a decision on the correct age group. For each face stimulus shown, the participants have to be able to discriminate one among ten age groups which represents the perceptual judgment of each participant. The strongest response denotes the signal and represents the actual age group while the remaining nine alternatives denote noise or uncertainty distributed among other response categories. The distance between the means of the signal and the noise distributions are compared against the standard deviation of the noise distribution to compute the discriminability index (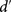) [28], [29]. The 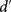 values calculated for each age group stimulus is shown in Table 2. Higher values of 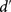 signify that the participants are able to discriminate a particular age group category better. From Table 2, the results show that participants were able to discriminate the two age group categories 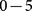 and 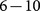 better than any other category and the 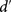 values for these correspond to 2.7500 and 2.3971 respectively. It is also observed that the 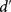 values for all age groups are positive representing that the responses obtained are better than random guesses.

The process of choosing a specific age group based on the visual stimulus presented depends on the information perceived in the stimulus by the participants. The perceived information can be quantitatively represented by the information entropy [30]. The perceptual information may have some residual uncertainty due to noise in the actual stimulus leading to incorrect predictions by the participants. The uncertainty is also introduced when the number of response categories are more. From the stimulus-response confusion matrix (Table 1), face stimulus entropy 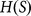 (Equation (1)) and noise or equivocation denoted by 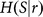 (Equation (2)) are calculated for each age group where 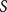 denotes the stimulus, 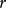 denotes the response of the participants, and 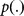 represents probability of respective terms. Information entropy 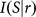 for each age group category is calculated by subtracting the noise, 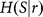 from the signal, 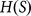 (Equation (3)).
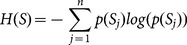
(1)

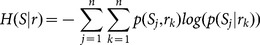
(2)

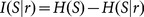
(3)


Dividing Equations (1) to (3) by 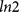, the values of the stimulus entropy, noise and information entropy for each age group are expressed in *bits* and are summarized in Table 2. Larger value of information entropy of an age group indicates that participants can accurately predict the stimulus belonging to that age group as the residual uncertainty is low. The results in Table 2 confirm that the two age groups 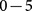 and 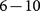 have the highest information entropy of 0.3171 bits and 0.2718 bits respectively.

Low values of accuracy for older age groups such as 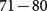 and >80 can be attributed to various factors which affect facial age progression of an individual. The factors including but not limited to gender, ethnicity, stress levels, dietary habits, and facial aging patterns of kin, combine to form a personalized facial age progression function for each person. Large variances in these factors may lead to incorrect perception of facial age by humans.

We also compared the human performance with an independently trained automatic algorithm. The same images are evaluated using Face++ [31], a face recognition tool built using deep face representation. An overall age group prediction of 42% is obtained on the same set of images. Upon further analysis, it is observed that images belonging to age groups of 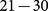, 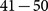, 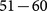, and 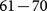 achieved only 20% accuracy which is lower than responses of human participants. Images belonging to 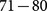 and >80 yielded an accuracy of 0% (none of the images in these age groups were correctly estimated). This suggests that there is a large scope for further improvement in current automated age prediction algorithms, especially if we are able to emulate the way humans perceptually estimate facial age.

### Group Bias in Age Group Estimation

In order to examine the existence of any group bias in age group estimation; ethnicity, gender and age group of the participants are compared with the stimuli ethnicity, gender and age group and the results are documented in Tables 3, 4 and 5 respectively.

**Table 3:** Effect of participant ethnicity and ethnicity of the presented face stimuli on age estimation accuracy.

Participant Ethnicity (# of participants)	Face Stimuli Ethnicity (Accuracy in %)	
	Indian	Caucasian
Indian (366)	30.88	**55.82**
Caucasian (81)	24.69	**57.71**

The results show that irrespective of the participant's ethnicity, the age estimation accuracy is higher for Caucasian face stimuli.

**Table 4:** Age group estimation accuracy based on participant's gender and face stimuli's gender.

Participant Gender (# of participants)	Face Stimuli Gender (Accuracy in %)	
	Male	Female
Male (201)	45.00	**52.81**
Female (281)	47.06	**57.78**

The results show that irrespective of the gender of the participants, the accuracy for female stimuli's are better.

**Table 5:** Age estimation accuracy based on participant's age and age of the face stimuli across various age groups.

Participant Age Group (# of participants)	Face Stimuli Age (Accuracy in %)			
	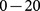	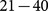	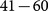	> 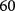
0–20 (14)	**66.67**	33.33	50.00	34.78
21–40 (385)	**69.58**	52.85	38.48	36.00
41–60 (66)	**78.84**	46.48	44.44	36.95
>60 (17)	**80.00**	41.17	26.67	30.43

The results show that participants from all age groups provide the best results on stimuli faces belonging to age group of 0–20.

The results in Table 3 show that Indian participants achieve an accuracy of 55.82% for Caucasian face stimuli while Caucasian participants are able to detect the age group of Caucasian faces with an accuracy of 57.71%. *Z*-test of proportions [32] is used for calculating if there is a significant difference in proportions of correct responses from any ethnicity of participants. At 95% confidence level, the results show that responses from any particular ethnicity are not significant, thus, validating the hypothesis of absence of any ethnicity based bias in age group estimation. In their review, Meissner and Brigham [33] showed that people are 1.4 times more likely to *identify* faces belonging to their own race and 1.56 times less likely to be falsely matched. As per our opinion, the above statement may only hold true for recognizing faces when there is no significant age variation, as the faces used in their study did not have any aging variations.

To examine if gender influences the judgment of perceived facial age, the gender of the participant is compared with the gender of the face stimuli. As shown in Table 4, male participants achieve an accuracy of 45% for male facial images and 52.81% for female faces whereas females participants yield 47.06% accuracy while judging male face stimuli and 57.78% while judging female faces. From these results it is evident that estimating female face stimuli is relatively easier compared to male faces. Both male and female participants are equal at predicting the age group of the female face stimuli images. *Z*-test of proportions at 95% confidence level also shows that gender of the participant does not act as a bias in the task of age group estimation. The result coheres with the observations made by Cross et al. [34] and Megreya et al. [35] in face recognition. They assert that female faces are recognized more frequently and the gender of the participant is not significant for performing the task of age group estimation.

It can be observed from Table 5 that individuals belonging to the age group 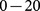 years are most easily estimated by the participants of all age groups. The participants belonging to the age group 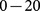 (Minimum age of participants in this group is 18) years, 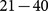, 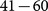, and >60 achieved 66.67%, 69.58%, 78.84%, and 80% accuracy respectively while classifying facial stimuli belonging to 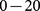 years age group. In this case, no influence of own-age estimation bias is observed. Similar to the previous results, test of proportions is performed for evaluating the validity of this hypothesis. The results obtained after applying *Z*-test of proportions [32] at 95% confidence level demonstrates that no age group of participants has significant effect on age group prediction performance. These findings are consistent with the observations by Burt and Perrett [6] where they deny the presence of own-age bias in age group estimation task.

### Effect of Facial Regions in Age Estimation

For understanding which facial region is most effective for estimating the age group of a given image, five facial regions are presented to the participants and are asked to estimate the age on the basis of a given facial region. As shown in Fig. 2, the five facial regions are T-region, T-region masked, binocular region, eyes portion masked, and chin-mouth region. The results for this experiment are presented in Table 6. It is observed that the information contained in the chin and mouth regions is sufficient to yield an accuracy of **100%** for infants and toddlers (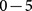 years age group). The reason for such a high accuracy is based on the fact that lower jaw region of individuals in this age group is significantly different from other age groups. With the T-region obfuscated, maximum correct responses are obtained for age group 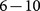, indicating that humans can show good performance if the features of the T-region for this age group are masked. Similar trend is also observed for age groups 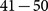 and 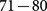. These results indicate that if one source of information (i.e. facial region) is occluded, the performance of age estimation is not completely degraded [9], [10].

**Table 6:** Analyzing the effect of facial regions in predicting the age group of the visual stimuli.

Stimuli Age Groups	Facial Region (Accuracy in %)				
	T Region	T-Region Masked	Binocular	Eyes Masked	Chin
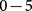	50.00	50.00	95.92	76.00	**100**
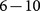	76.00	85.42	26.00	77.08	68.75
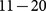	56.09	32.00	59.09	63.26	23.68
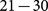	51.02	22.22	54.00	35.42	42.00
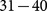	32.00	40.81	45.83	50.00	46.81
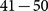	39.13	55.55	22.22	39.13	39.02
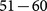	48.84	24.49	24.49	33.33	41.67
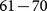	46.81	56.01	36.36	57.14	54.00
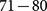	12.50	53.48	38.09	26.83	14.58
> 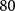	11.90	19.04	20.83	20.93	20.93

The results show that chin region of children belonging to age group 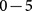 are most discriminating for age prediction.

### Face Recognition across Age Progression

After assessing the ability to estimate the age group, the next step is to understand how efficient humans are in recognizing age-separated images of an individual. As shown in Fig. 3, the participants are presented with a pair of age-separated images and they are asked to determine if the two images belong to the same individual. The results are summarized in Table 7. The column *Stimuli Age Groups* represents the age group of the two presented images.

**Table 7:** Face recognition accuracy achieved with respect to stimuli age group and type of facial region shown.

Stimuli Age Groups	Facial Region (Accuracy ± Standard Deviation in %)				
	Full Face	Binocular	T Region	T-Region Masked	Chin
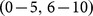	60.41±1.13	**67.02±0.27**	59.37±2.10	33.33±0.14	50.55±0.13
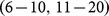	**81.52±0.01**	69.47±2.11	76.59±0.91	69.38±0.04	66.67±2.61
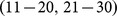	**87.00±0.38**	68.89±3.00	67.34±1.07	65.21±0.02	43.75±0.3
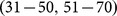	**76.53±2.31**	54.08±0.45	63.33±1.71	57.14±1.01	59.13±1.26
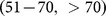	70.83±0.98	55.10±0.42	72.00±0.36	66.30±1.22	**80.61±0.02**

The values in bold show which region is the most discriminating for recognizing the stimuli belonging to a given age group. It can be observed that in general, the whole face yields the highest accuracy whereas for children and elderly people, binocular and chin regions provide the most discriminating features respectively.

On analyzing the accuracies for various age group pairs, it is observed that it is more challenging to identify individuals during the formative years of their lives. The row (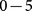, 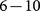) of Table 7 shows that the accuracy obtained for these image pairs belonging to the two age groups is lower compared to any other age group. For this pair, the maximum accuracy of 67.02% is achieved for binocular region. This is the least among the maximum accuracies obtained by all the age group pairs. The results indicate that during this time period, the face of an individual undergoes a significant amount of variations leading to difficulty in recognizing age-separated images. The best performance of 87% is attained when the pair of images belong to age category (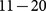, 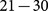).

It can be seen that for majority of the cases, the maximum accuracy is obtained when the presented pair of images contains full face of the individual, signifying that humans use the information present in the entire face for recognizing people. *Z*-test of proportions [32] at 95% confidence level, also supports this claim. It is also observed that the binocular region for age groups (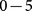, 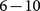) contains invariant features which are required for recognition. In this scenario, the participants achieve an accuracy of 67.02%. Similar performance is observed when the participants are shown age-separated images of lower facial (chin) region belonging to age groups (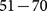 and >70). In order to compare the performance of human evaluation with an independently trained algorithm, the pairs of face image stimuli are evaluated using Face++ [31]. Using the same experimental setting, this tool yields verification accuracy of 60% at Equal Error Rate (EER) of 40%. It is observed that when the age gap between the images is high or one of the images belongs to the childhood of the subject, Face++ yields incorrect output. To overcome this weakness, the results suggest that machine learning algorithms can incorporate cues from human perception and improve the accuracy of current face recognition systems.

## Face Recognition Algorithm Inspired from Human Analysis

An important component of this study is to demonstrate that the knowledge learned from human observations can be utilized for improving a face recognition algorithm to address age variations. One possible approach to incorporate the knowledge is:

- Estimate the age of the probe image.
- Extract facial regions such as binocular, T-region, T-region masked, and chin regions using facial key points and golden ratio template. This step is followed by extraction of features such as texture features using local binary patterns [36] for each facial region.
- For matching a gallery image with the probe image, assign weights to various facial regions based on the predicted age and the relevance of that facial region for age-separated face recognition using Table 7.

**Figure 4:**
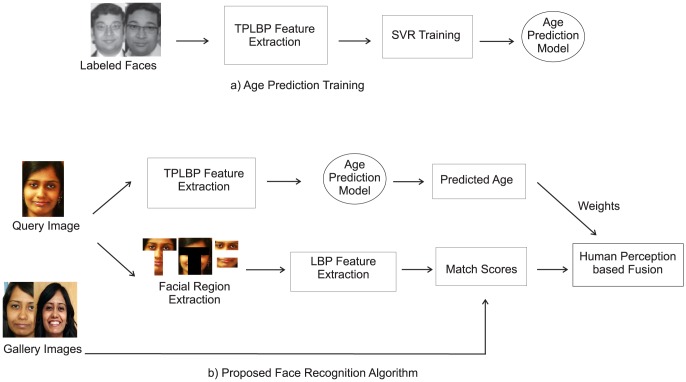
Proposed human perception based fusion scheme (HPFS) for age prediction and face recognition.

We term this approach as **human perception based fusion scheme (HPFS) for face recognition** (Fig. 4). In this approach, we use existing feature extractors to demonstrate that incorporating the knowledge gained from human analysis (in terms of weights) can significantly enhance the performance. The details of age estimation and face recognition algorithm are discussed in subsequent subsections.

### Facial Age Group Estimation using Three Patch Local Binary Patterns (TPLBP)

The objective of this part is to design an age group estimation function, trained by a set of labeled faces (age group being the label), that can estimate the age of any given query face. For this purpose, a variant of LBP termed as Three Patch LBP (TPLBP) [37], is used as the feature descriptor. The high efficiency of TPLBP in face recognition shows that it can efficiently encode discriminating facial features. In TPLBP, for each pixel, 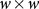 size patches are selected for comparison. Two such patches at a distance of 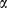 from the center pixel are compared to set the bit value.

Given a labeled training set of faces, TPLBP descriptor is extracted for each of the faces. This descriptor of the training set is given as input to the Support Vector Regressor (SVR - Support Vector Machine in Regression Mode) for implementing an age prediction function. Kernel testing [38] is performed in order to obtain the best parameters of the SVR trained model. Once trained, the algorithm predicts the age group of the given input probe face image.

### Human Perception based Fusion Scheme for Face Recognition

After estimating the age group of the query image, the next task is to match the identity of the image with gallery (database) images. The face matching algorithm is explained below:

1. **Face Parts Extraction:** Full face, binocular region, T-region, T-region masked, and chin region are obtained for the input image using facial landmark detection.
2. **LBP Calculation:** For each of the facial region, uniform circular LBP features [36] are extracted. These features are matched with the corresponding gallery features and match scores are obtained pertaining to each of the five facial regions.
3. **Score Fusion via Learned Weights:** The five match scores corresponding to each face recgion are combined using score fusion [39]. The simplest approach is to apply sum rule i.e., 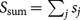, where 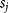 is the match score pertaining to the 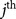 region. The fused score can then be used for matching. To enhance the performance, weighted sum rule is used [39] i.e., 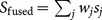, where 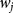 is the weight pertaining to the 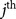 region. The weight 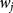 can be obtained empirically using the accuracy of individual facial regions.

In this research, we compute the weights learned from the accuracies obtained from human performance evaluation. Using Table 7, the weights of the proposed human perception based sum rule fusion scheme are calculated as follows: 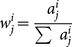
(4)


Let 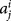 be the accuracy of the 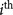 age group of the gallery and probe and the 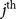 facial region where 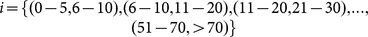 (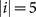) and 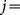
{full-face, binocular, T-region, T-region masked, chin}. The weights for weighted sum rule are computed using Equation (4). These weights are then used to compute the final score using Equation (5). In this equation, 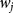 represents the weight calculated in Equation (4) and 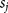 represents the matching score corresponding to the facial regions.
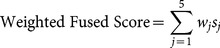
(5)


If the age of the individual in the gallery lies in the age group 0–5 years and the predicted age of the probe image comes out to be in the range of 6–10 years, then using row 1 of Table 7 (learned via human evaluation), we can assign weights to the facial regions based on the obtained accuracies. These weights are then used for weighted sum rule fusion. For the gallery and probe pairs, where the age gap is greater than those mentioned in Table 7, sum rule fusion is applied to LBP scores of all the facial regions. The fused score is finally used as match score to recognize the face image.

### Algorithm Evaluation

Three datasets are used for evaluating the performance of the above described approach: IIIT-Delhi facial aging database [24], [25], FG-Net Aging Database [23], and MORPH Album1 [40] database. The IIIT-Delhi facial aging database consists of over 2600+ age-separated labeled face images of 102 individuals (Indian celebrities) in the age range of 4–88 years. The FG-Net database contains 1002 age-separated face images of 82 subjects. The age of the subjects in the database ranges from 0 to 69 years. On average, there are 12 images per subject in the database. MORPH Album1 contains 1,690 scanned images of 515 subjects. The age of the subjects ranges from 15–68 years.

All the datasets are divided into two partitions, approximately 30% of the subjects are used for training and the remaining unseen 70% for testing. The training partition is used for training the SVR model for age estimation. Two experiments [25] are performed on the databases. In the first experiment, the probe set consists of one among the latest (oldest) face images of every subject, while the remaining images are in the gallery. In the second experiment, the probe set contains one earliest (youngest) face image of every subject and the remaining images are used as gallery. These experimental protocols are used as the age variation is maximum in these two scenarios. Hence, it would be necessary to evaluate the performance of the proposed algorithm in these cases. The age of probe images is estimated using the trained SVR model. Subject to the age of the gallery and probe subjects, appropriate weights are assigned to the facial regions based on their relevance in age-separated face recognition. The results of the proposed approach are compared with VeriLook [41] (referred as COTS: Commercial Off-The-Shelf), Face++ [31], and other fusion approaches, namely sum rule [39], [42], weighted sum rule [39], [42], and SVM fusion [43]. The performance is also compared with Discriminative Model (DM)-based face recognition algorithm [20] for facial aging. Table 8 summarizes the results of the proposed and existing recognition algorithms.

**Table 8:** Comparing the face recognition accuracy of the proposed (HPFS) algorithm and existing algorithms using IIIT-Delhi, FG-Net Aging, and MORPH databases.

Algorithm	Facial Region(s)	Oldest Image as Probe			Youngest Image as Probe		
		IIIT-Delhi	FG-Net	MORPH	IIIT-Delhi	FG-Net	MORPH
(1) Unimodal	Face	26.1%	10.0%	4.2%	25.0%	3.4%	14.2%
(2) Sum Rule	Face, Mouth	21.1%	10.3%	15.2%	13.9%	3.4%	12.3%
(3) Sum Rule	Face, Binocular	25.7%	17.6%	11.3%	19.6%	6.5%	12.0%
(4) Sum Rule	Periocular	26.1%	19.8%	9.3%	19.6%	4.3%	7.6%
(5) Sum Rule	Binocular, Periocular	26.4%	20.2%	9.3%	20.0%	5.6%	7.2%
(6) Sum Rule	Face, Periocular	30.4%	19.8%	11%	21.1%	7.7%	12.3%
(7) Sum Rule	Face, Binocular, Mouth	26.8%	14.6%	13.7%	18.9%	5.6%	15.6%
(8) Sum Rule	Mouth, Periocular	29.0%	19.8%	13.5%	21.8%	6.5%	12.8%
(9) Sum Rule	Face, Binocular, Periocular	29.6%	20.2%	14%	22.5%	8.6%	12.8%
(10) Sum Rule	Face, Mouth, Binocular, Periocular	31.8%	18.5%	16.3%	21.8%	7.3%	15.8%
(11) Weighted Sum	Face, Mouth, Binocular, Periocular	32.8%	20.7%	22.3%	22.1%	9.5%	17.4%
(12) SVM Fusion	Face, Mouth, Binocular, Periocular	8.6%	4.2%	3.6%	10.0%	2.3%	6.5%
(13) DM [20]	Overlapping Patches	52.8%	62.7%	21.3%	30.4%	28.0%	14.9%
(14) Face++ [31]	Face	48.2%	55.7%	17.2%	29.4%	21.6%	18.5%
(15) VeriLook (COTS)	Face	52.7%	51.6%	15.2%	27.8%	8.9%	12.1%
(16) Proposed Human Perception based Fusion Scheme (HPFS) with *Actual* Age Groups	Face, Binocular, T-Region, Not T-Region, Chin	45.3%	51.0%	26.2%	39.6%	31.4%	29.9%
(17) **Proposed Human Perception based Fusion Scheme (HPFS) with ** ***Predicted*** ** Age Groups**	**Face, Binocular, T-Region, Not T-Region, Chin**	**42.9%**	**45.9%**	**22.5%**	**34.3%**	**28.4%**	**21.5%**

The results of the proposed human perception based estimation and recognition algorithm is shown in bold.

### Analysis

#### Results on IIIT-Delhi Facial Aging Dataset

For the IIIT-Delhi facial aging dataset, the proposed fusion rule HPFS (Row 17 in Table 8) outperforms most of the existing algorithms. On comparing with the commercial system (COTS) (Row 15), Face++ [31] (Row 14), and DM [20] (Row 13), the proposed algorithm yields higher accuracy when the probe images belong to the youngest image of the subjects. Rank-1 accuracy of the proposed approach is 34.3% whereas the COTS, Face++ [31] and DM [20] yield 27.8%, 29.4%, and 30.4% respectively. This technique is useful in locating children who have been kidnapped when they were young. Images from their childhood can be kept in probe while current images of people can be in the gallery for finding if a match exists. For the experiment where the probe set consisted of the oldest images of the subjects, an accuracy of 42.9% is achieved. This is higher than traditional fusion schemes such as sum rule, weighted sum rule, and SVM fusion but lower than COTS, Face++ [31], and DM [20].

#### Results on FG-Net Aging Dataset

Similar to the results on IIIT-Delhi Facial Aging dataset, the results obtained on the FG-Net aging database suggest that the proposed HPFS (Row 17) outperforms traditional fusion schemes for both sets of experiments. However, for experiments using the oldest images as probe, COTS (Row 15), Face++ [31] (Row 14), and DM [20] (Row 13) outperform the proposed approach. On the other hand, for experiments using youngest images as probe, the performance of the proposed approach is the best (i.e. 28.4%).

#### Results on MORPH Dataset

From Table 8, on the MORPH database, it can also be seen that the proposed approach outperforms the traditional fusion approaches, DM [20] (Row 13), Face++ [31] (Row 14), and COTS (Row 15) for both the experiments. For the experiment where the probe set contains the latest images of the subjects, an accuracy of 22.45% is obtained while for the experiment where the youngest images of the subjects are in the probe, rank-1 accuracy of 21.54% is achieved.

#### Error Produced by Age Estimation

In order to highlight the error produced by the age estimation algorithm used in our proposed approach, Table 8 (Row 16) contains results from the scenario if actual age groups (ground truth) of the probe images are used, instead of age prediction in the proposed Human Perception based Fusion Scheme algorithm. It can be seen that if a good age estimation algorithm is developed in the future, it can be incorporated in our approach to obtain significant increase in the performance.

It should be noted that the objective of these experiments is to illustrate that using existing facial feature extraction approach, the human perception based scheme can improve the performance of face recognition with aging variations. It is our assertion that the same framework can be applied to other facial feature extraction algorithms as well and similar improvements may be observed. However, there may be other ways to incorporate these findings/observations in an automatic face recognition algorithm.

## Limitations of the Study and Open Questions

There are a few limitations of our research and some questions are yet to be explored. The proposed algorithm for age-invariant face recognition yields lower results that Face++ [31], COTS and DM [20] for *oldest image as probe* experiment on the IIIT-Delhi and FG-Net databases. Currently, we are extending the algorithm to further improve the performance, specifically with both age and weight variations [44]. An inherent problem in studying the process of facial aging is the lack of data. It is very difficult to collect images for all the subjects for all the age groups. If more samples per age group per person are available, then a finer granularity in weight computation can be performed. The study can also be extended on some other publicly available facial aging database. Further, there is a need to develop a better age group estimation algorithm to further boost the performance of the proposed approach. It would be interesting to study the human performance for various age estimation tasks if there is more diversity in the demographics of the participants.

## Conclusions

Faces undergo significant variations during the lifetime on an individual. This research attempts to analyze how humans perceive facial age and their ability to estimate age. The results indicate that age estimation for newborns and toddlers is easiest and a person's gender or ethnicity does not affect the performance of age group estimation. The research presents the effect of facial regions such as binocular region, T-region, and mouth on the age prediction accuracy. As a global feature, full face achieves good performance in age-separated face recognition. Using selected feature cues gathered from the research, we propose the human perception based weighted score fusion rule to enhance the face recognition accuracy with age variations. The proposed algorithm demonstrates improvement in accuracy on three facial aging databases when compared with existing approaches and commercial face recognition systems.
